# Supplementary figures and images for: Gastric Carcinomas with Stromal B7-H3 Expression Have Lower Intratumoural CD8+ T Cell Density
Source: Int J Mol Sci. 2021 Feb 21;22(4):2129. doi: 10.3390/ijms22042129 (PMC7924590; doi:10.3390/ijms22042129)

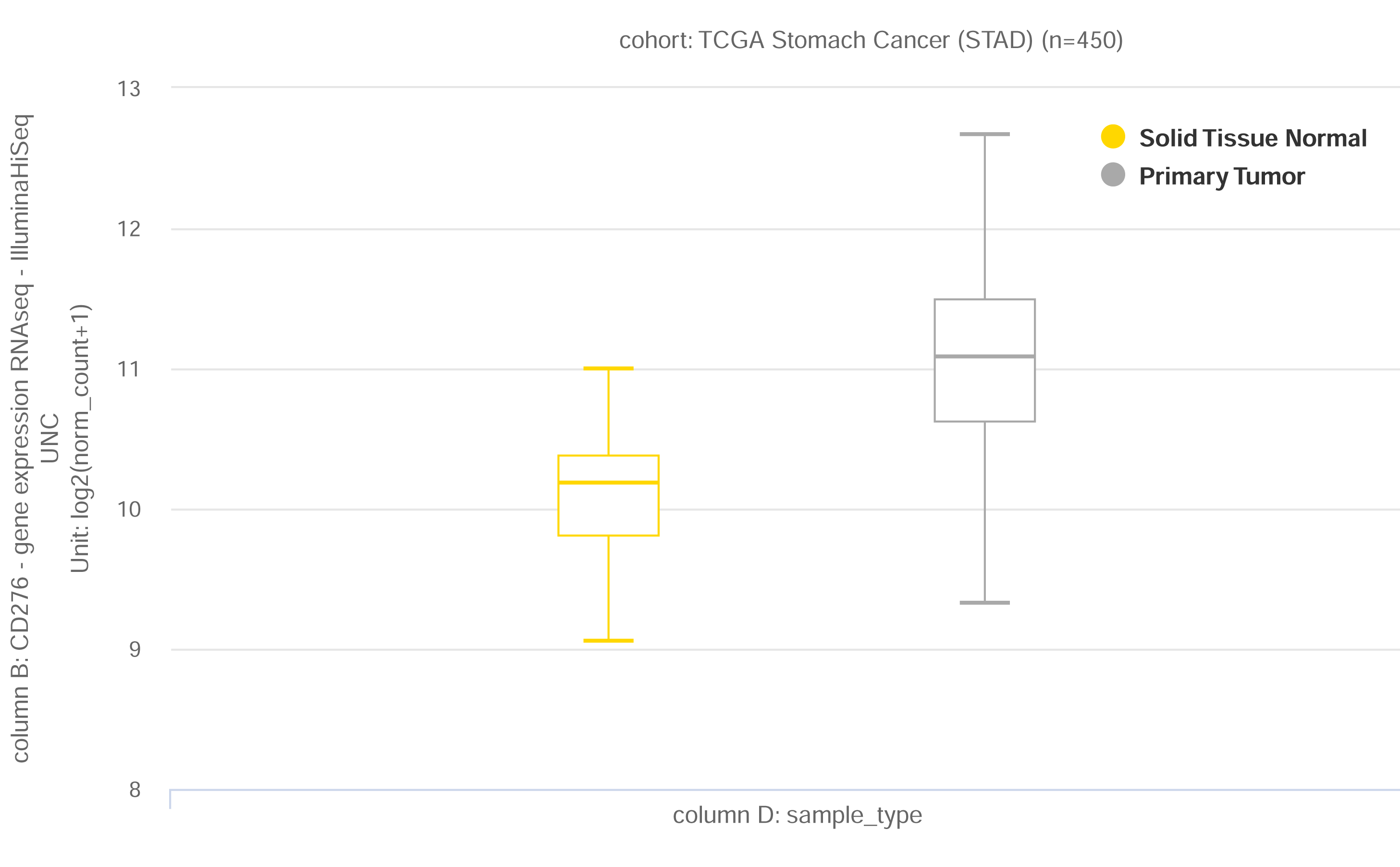

Supplement: Supplementary file 1 [file ijms-22-02129-s001.zip › Figure S1_tcga_stad.tif]

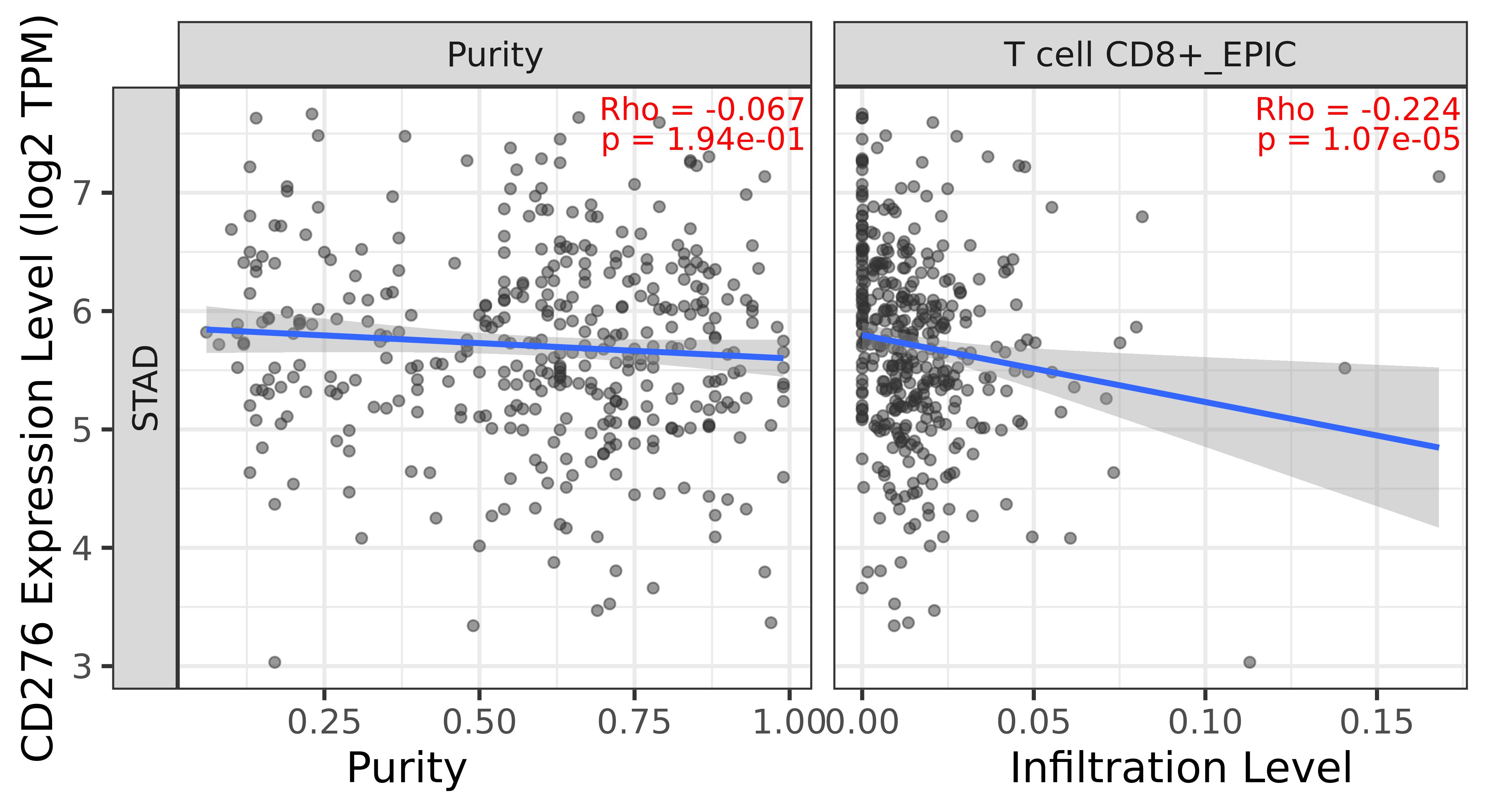

Supplement: Supplementary file 1 [file ijms-22-02129-s001.zip › Figure S2_gene_plot.jpg]
